# Assay-dependent variability in peptide biomarker quantification: experimental evidence from renalase in chronic kidney disease

**DOI:** 10.1080/07853890.2026.2668217

**Published:** 2026-05-12

**Authors:** Natalia Serwin, Elżbieta Cecerska-Heryć, Bartłomiej Grygorcewicz, Magda Wiśniewska, Aleksandra Gomółka, Katarzyna Bąk, Aleksandra Cader-Ptak, Bartosz Wojciuk, Marcin Lisak, Martyna Opara-Bajerowicz, Aleksandra Polikowska, Małgorzata Goszka, Karol Serwin, Mariusz Suwała, Katarzyna Fiedorowicz, Kazimierz Ciechanowski, Barbara Dołęgowska

**Affiliations:** ^a^ Department of Laboratory Medicine, Pomeranian Medical University in Szczecin, Szczecin, Poland; ^b^ Department of Genomics and Forensic Genetics, Pomeranian Medical University in Szczecin, Szczecin, Poland; ^c^ Department of Nephrology, Transplantology, and Internal Medicine, Pomeranian Medical University in Szczecin, Szczecin, Poland; ^d^ Department of Immunological Diagnostics, Faculty of Medicine, Pomeranian Medical University in Szczecin, Szczecin, Poland; ^e^ Department of Infectious, Tropical Diseases, and Acquired Immunodeficiency, Pomeranian Medical University in Szczecin, Szczecin, Poland; ^f^ Department of Periodontology, Pomeranian Medical University in Szczecin, Szczecin, Poland

**Keywords:** Renalase, chronic kidney disease, ELISA assay variability, biomarker variability, assay standardisation, matrix effect

## Abstract

**Background:**

Renalase is a promising biomarker for kidney disease, but published levels vary widely between studies. We hypothesised that variability in commercial enzyme-linked immunosorbent assays (ELISAs) kits and matrix effects (serum vs plasma) drive these inconsistencies.

**Methods:**

Paired serum and plasma samples from 56 participants (28 chronic kidney disease (CKD) stages 2–5, 28 healthy controls) were tested using three commercial renalase ELISAs (BTLAB, Cloud-Clone, EIAab). We assessed intra-assay precision, inter-assay agreement (Spearman’s rank correlation and Bland–Altman analysis on log10-transformed values), matrix effects, and associations with estimated glomerular filtration rate (eGFR). Diagnostic performance was evaluated by Receiver operating characteristic (ROC) analysis.

**Results:**

Inter-assay renalase concentrations differed markedly (up to orders of magnitude), with weak inter-assay correlations (*r* ≤ 0.25). Bland–Altman analyses revealed large, systematic biases between kits. Only the BTLAB assay showed consistent serum/plasma agreement, a significant correlation with eGFR (ρ ≈ 0.32–0.42, *p* < 0.05), and moderate discriminatory performance for CKD in serum (AUC = 0.70) and plasma (AUC = 0.68). Cloud-Clone and EIAab produced divergent results and strong matrix-dependent biases.

**Conclusions:**

Observed variability among commercial ELISA platforms may compromise comparability between studies. Harmonisation, standardised reference materials, and cross-validation are necessary before renalase assays can be used reliably in clinical practice.

## Introduction

Renalase, a flavoprotein secreted primarily by the kidneys and other metabolically active tissues, has emerged as a molecule of significant interest in nephrology due to its proposed roles in cardiovascular regulation, catecholamine metabolism, and cellular stress response pathways. Initially identified for its ability to degrade circulating catecholamines and modulate blood pressure and sympathetic tone, subsequent research has expanded its putative functions to include anti-inflammatory and cytoprotective effects, particularly in the context of ischemia-reperfusion injury and chronic kidney disease (CKD) [1,2].

Despite a growing body of literature implicating renalase in renal pathophysiology, the accurate and consistent quantification of circulating renalase remains a major challenge. Numerous studies have attempted to measure serum or plasma renalase levels across various disease states—including hypertension, CKD, diabetes, and cardiovascular disease—but with strikingly heterogeneous results, with reported concentrations differing by several orders of magnitude across studies, sometimes reaching differences of tens- to hundreds-fold even in apparently similar clinical settings [3–10]. In addition to methodological variability, the biological complexity of circulating renalase may also contribute to these discrepancies. Chang et al. [11] demonstrated the presence of distinct, measurable forms of renalase in human plasma, suggesting that the protein’s molecular heterogeneity can influence immunoassay detection and clinical interpretation. These discrepancies are often attributed to differences in sample type, cohort characteristics, and, critically, the use of distinct immunoassay platforms, primarily enzyme-linked immunosorbent assays (ELISAs).

ELISA tests were developed in the 1970s as a faster, more cost-effective alternative for detecting antigens and antibodies compared with older methods [12,13]. The technique relies on an enzyme linked to an antibody that produces a measurable signal, enabling the detection of specific substances in biological samples. ELISA methods have been used for decades to identify both commonly known substances and newly discovered molecules. In the scientific literature, the issue of limited standardisation among commercially available ELISA kits has been repeatedly emphasised [14]. Even when different kits are nominally “specific” for the same protein, variations in intrinsic antibody affinity, differences in epitope recognition associated with the use of monoclonal versus polyclonal antibody systems, and diversity in detection formats (e.g. chromogenic enzyme-based versus fluorescent readouts) may contribute to substantial discrepancies in reported concentrations [15,16]. For example, in a study by Quinn et al., IL-6 levels measured by a magnetic multiplex assay differed significantly from those obtained with high-sensitivity ELISA [17], highlighting how assay platform choice can lead to substantial variability in cytokine quantification. This issue closely parallels the discrepancies observed in renalase measurements across ELISA kits.

Although ELISA remains a widely employed immunoassay technique, it has several well-recognized technical limitations. These include: strong dependence of results on sample type (serum vs. plasma), matrix effects caused by serum proteins such as albumin or immunoglobulins, substantial inter-laboratory and inter-assay variability [18], susceptibility to protein degradation or improper sample storage conditions [19]. The reproducibility of ELISA-based measurements—intra- and inter-assay precision—is critical for clinical reliability. Studies involving other biomarkers have shown that acceptable CVs for ELISA in clinical diagnostics should not exceed 10–15% [20].

Commercial ELISA kits from different manufacturers have been widely used for renalase quantification; however, they are often designed with proprietary antibodies targeting different epitopes, employ variable calibrators, and lack harmonised reference standards. As a result, reported renalase concentrations can vary by several orders of magnitude across studies [3–10], raising significant concerns about assay specificity, cross-reactivity, and reproducibility. For example, some studies have reported elevated circulating renalase levels in patients with CKD, whereas others have observed reduced or unchanged concentrations, even in clinically comparable renal or cardiovascular populations [5,21–24]. A detailed overview of previously published findings, including study populations, biological matrices, and assay manufacturers, is presented in Table 1. This methodological heterogeneity complicates the interpretation of renalase as a biomarker and limits its integration into clinical practice. Without assay standardisation and rigorous validation, it remains unclear whether observed variations in renalase levels reflect true biological differences or are artefacts of assay performance. Therefore, a critical evaluation of assay comparability is essential to advance renalase research and assess its utility as a diagnostic or prognostic indicator in nephrology.

**Table 1.** Renalase concentrations in study groups: CKD (including HD, PD), kidney transplant recipients (kTx), arterial hypertension (AH), coronary artery disease (CAD), heart transplant recipients (hTx), patients after surgical repair of aortic coarctation (CoA), and healthy controls (CON).

Group	Additional criteria/group description	Material	Results	ELISA manufacturer	Ref.
Patients at various stages of chronic kidney disease (CKD), including those undergoing peritoneal dialysis (PD) or hemodialysis (HD).	CKD: *N* = 139, including: St. I/II: 30, St. III: 30, St. IV: 30, St. V (haemodialyzed): 49 control: *N* = 45	Serum	Control: 251.0 ± 157.0 ng/mL CKD: 316.1 ± 155.3 ng/mL, control vs CKD *p* < 0.026 Stage I/II: 354.5 ± 181.6 ng/mL Stage III: 253.3 ± 152.3 ng/mL Stage IV: 279 ± 162.2 ng/mL Stage V (dialyzed): 369.0 ± 110.5 ng/mL *p* < 0.0001 (st. V vs st. I/II)	USCN Life Science Inc, Wuhan, China	[3]
	CKD: *N* = 87 (49M/38K) including: St. I: 8, St. II: 19, St. III: 15, St. IV: 8, St. V: 37 (7 non-dialyzed, 21 on HD, 9 on PD) control: *N* = 15 Underlying conditions: glomerulonephritis (GN), diabetic nephropathy, hypertensive nephropathy, autosomal dominant polycystic kidney disease (ADPKD), gouty nephropathy.	Serum	CKD St. I-II: 162.1 ± 40.1 ng/L CKD St. III-V: 217.4 ± 103.8 ng/L control: 167.8 ± 69.4 ng/L control vs St. I-II: *p* > 0.05 St. I-II vs St. III-V: *p* < 0.05	Yaji Biological Corporation, Shanghai, China	[4]
	CKD: *N* = 155 control: *N* = 30	Serum, erythrocytes	Serum: CKD: 143.1 ± 194.9 ng/mL Control: 19.6 ± 5.0 *p* < 0.001 Erythrocytes (ng of renalase per 1 g of Hb): CKD: 464.0 (382.4) ng/mL Control: 697.6 (273.4) ng/mL *p* < 0.001	EIAab, Wuhan, China	[21]
	CKD: *N* = 62 control: *N* = 28	Serum, urine	Serum: control: 11.1 (2.5–26.5) ng/mL CKD: 36.1 (18.3–109.1) ng/mL *p* < 0.001 Urinary renalase/Cr ratio (ng/mg): CKD: 44 (17.7–172) ng/mg control: 53.7 (22.7–96.5) ng/mg *P* = 0.99	EIAab, Wuhan, China	[24]
	CKD: *N* = 90 including: st. III: 30; st. IV: 30; st. V (haemodialyzed): 30 control: *N* = 30	Serum	control: 21.8 ± 9.2 ug/mL St. III: 20.2 ± 3.1 µg/mL St. IV: 24.9 ± 4.1 µg/mL HD: 35.6 ± 13.5 µg/mL significant differences: control vs HD CKD St. III vs HD CKD St. III vs St. IV CKD St. IV vs HD	CloudClone Corp, Houston, TX, USA	[5]
	CKD: *N* = 117 including: HD: 32; DO: 31; CKD: 30; Tx: 24; control: *N* = 31	Plasma	Before Hemodialysis (HD A): 5709 ± 7930 ng/mL After Hemodialysis (HD B): 7944 ± 7285 ng/mL Peritoneal dialysis (PD): 9946 ± 7858 ng/mL Conservative treatment (CKD): 8224 ± 7526 ng/mL Before kidney transplant (TE A): 11 331 ± 9872 ng/mL After kidney transplant (TE B): 9464 ± 8291 ng/mL Control Group: 5658 ± 5264 ng/mL *p* < 0.001	CloudClone Corp, Houston, TX, USA	[25]
	HD: *N* = 49 (M)	Plasma	HD: 26.59 ± 32.63 ng/mL	USCN Life Science Inc.	[26]
	HD: *N* = 50 control: *N* = 35	Serum	HD: 212 ± 127 ng/mL control: 116 ± 67 ng/mL *p* < 0.001	N/D	[27]
	HD: *N* = 60 including: HD-DM(+): *N* = 19 HD-DM(−): *N* = 41 HD-AH(+): *N* = 24 HD-AH(−): *N* = 36 control: *N* = 16	Serum, urine	HD: 27.53 ± 9.39 µg/mL; control: 4.00 ± 1.37 µg/mL *p* < 0.01 HD-DM(+): 25.85 ± 9.17 µg/mL, HD-DM(−): 28.76 ± 6.26 µg/mL HD-AH(+): 26.98 ± 6.98 µg/mL p > 0.05 HD-AH(−): 30.11 ± 8.34 µg/mL *p* > 0.05	USCN Life Science Inc, Wuhan, China	[28]
	HD: *N* = 104, including: a) *N* = 65 with elevated blood pressure (>140/90 mmHg before dialysis and >130/80 mmHg after dialysis), b) *N* = 52 with residual kidney function and *N* = 52 without residual kidney function control: *N* = 27	Serum	HD: 27.53 ± 7.1 µg/mL; control: 3.86 ± 0.73 µg/mL, *p* < 0.001 HD: men vs women: 29.8 ± 7.28 vs 25.92 ± 7.79 µg/mL (*p* < 0.05)	USCN Life Science Inc, Wuhan, China	[29]
Patients with a simple renal cyst (SRC)	SRC: *N* = 75 control: *N* = 51	Serum	SRC: 130.3 ± 60.6 ng/mL control: 160.4 ± 57.6 ng/mL *p* < 0.05	Sunred Biological Technology Co., Ltd	[30]
Patients after unilateral or bilateral nephrectomy	Pediatric patients (<18 years old) with a single kidney (SK) SK: *N* = 36 control: *N* = 57	Serum, urine	SK: serum 23.07 µg/mL (19.96, 27.22), urine: 137.68 ng/mg Cr (96.05, 239.43) control: serum 26.75 µg/mL (22.64, 29.20) urine 187.93 ng/mg Cr (110.45, 286.66) serum *p* = 0.04 urine *p* = 0.64	USCN Life Science Inc., Wuhan, China	[31]
	HD: *N* = 100 HDF: *N* = 17 control: *N* = 24	Serum	HD: 25.98 ± 8.12 µg/mL HDF: 17.87 ± 6.91 µg/mL control: 3.98 ± 0.79 µg/mL HD vs HDF: *p* < 0.05 HD & HDF vs CON: *p* < 0.001	USCN Life Science Inc., Wuhan, China	[22]
Patients after kidney transplantation	kTx: *N* = 50	Serum	kTx: 6.6 ± 2.78 µg/mL	USCN Life Science Inc, Wuhan, China	[32]
	kTx: *N* = 89 control: *N* = 27	Serum	kTx: 6.72 ± 4.50 µg/mL; control: 3.86 ± 0.73 µg/mL; *p* < 0.001	USCN Life Science Inc, Wuhan, China	[33]
	kTx: *N* = 62 control: *N* = 27	Serum	control: 3.86 ± 0.73 μg/mL kTx: 6.72 ± 2.86 µg/mL *p* < 0.05	USCN Life Science Inc, Wuhan, China	[34]
	kTx onors (living): *N* = 20 kTx recipients: *N* = 20	Serum	Donors: before kTx: 125.2 ± 35.0 μg/mL After kTx: 140.2 ± 73.6 μg/mL *p* = 0.0004 Recipients: Before kTx: 242.4 ± 147.0 μg/mL After kTx: 162.3 ± 53.0 μg/mL *p* = 0.003	Eastbiopharm Co Ltd, Hangzhou, China	[35]
Patients with primary hypertension	*N* = 211, including: <65 y.o. *N* = 131 >65 y.o. *N* = 80	Serum	<65 y.o. 13.14 µg/mL >65 y.o. 20.59 µg/mL *p* = 0.02	USCN Life Science Inc., Wuhan, China	[6]
	AH: *N* = 121 control: *N* = 27	Serum	AH: 9.57 µg/mL (0.04, 62.5) control: 3.83 µg/mL (2.6, 5.3) *p* = 0.0001	USCN Life Science Inc., Wuhan, China	[36]
	Patients after successful surgical repair of aortic coarctation (CoA) using the Dacron patch technique *N* = 50 (31 males / 19 females) Also divided into subgroups: CoA + AH (with postoperative hypertension) CoA–AH (without hypertension) control: *N* = 50	Plasma	CoA: 5825.1 ng/mL; control: 6592.7 ng/mL *p* < 0.05 CoA + AH: 4946 ± 1786 ng/mL CoA-AH: 6461 ± 3607 ng/mL *p* = 0.03	USCN Life Science Inc., Wuhan, China	[8]
Patients with coronary artery disease (CAD)	Patients after coronary artery angiography CAD: *N* = 164 control: *N* = 140	Plasma	CAD: 112.50 ± 12.87 ng/L control: 120.79 ± 14.39 ng/L *p* < 0.05	Shanghai Boyao Biotechnology Co., Ltd	[7]
	Patients before and after coronary angiography/percutaneous coronary intervention, with preserved kidney function *N* = 95 (66 males, 29 females)	Urine	Baseline renalase concentration: 2843.6 ng/mg Cr 6 hours after the procedure: 1540.7 ng/mg Cr	Cloud-Clone Corp, Houston, USA	[37]
Patients after heart transplantation (hTx)	hTx: *N* = 80 control: *N* = 22	Serum	control: 3.86 ± 0.73 µg/mL hTx: 8.79 ± 4.85 µg/mL *p* < 0.05	USCN Life Sciences Inc, Wuhan, China	[38]
	hTx: Patients after heart transplantation (OHT – heart allograft recipients): *N* = 130 control: *N* = 27	Serum	control: 3.86 ± 0.73 µg/mL OHT: 8.41 ± 5.47 µg/mL *p* < 0.001	USCN Life Science Inc, Wuhan, China	[39]
	hTx: *N* = 130 control: *N* = 27	Serum	NYHA classification: I: 7.42 ± 4.18 μg/mL II: 10.59 ± 7.19 μg/mL *p* < 0.01 III: 12.86 ± 8.08 μg/mL CKD stages: St. 1: 4.57 ± 0.83 μg/mL; St. 2: 5.97 ± 1.79 μg/mL (*p* < *0.01* vs. st. 1) St. 3: 7.64 ± 2.67 μg/mL (*p* < *0*.01 vs st. 2 oraz *p* < *0.001* vs St. 1); St. 4: 12.17 ± 4.84 μg/mL *(p* < *0.001* vs. st. 3); St. 5: 27.69 ± 8.84 μg/mL *(p* < *0.001 vs* st. 4).	USCN Life Science Inc., Wuhan, China	[40]

NYHA: New York Heart Association.

Building on previous studies addressing biochemical heterogeneity of renalase, the present work focuses on the analytical consequences of assay selection under standardised laboratory conditions. In this study, we sought to systematically compare the analytical performance of three commercially available ELISA kits for renalase quantification. By analysing paired serum and plasma samples from patients with and without CKD, and correlating renalase levels with clinical parameters, we aimed to assess assay agreement, matrix effects, and diagnostic accuracy. Our findings highlight the need for methodological rigour and standardisation in renalase measurement and guide future clinical and translational applications of this promising biomarker.

## Methods

### Participants

The study was conducted between March 2023 and September 2024. It involved 56 participants − 28 individuals without chronic kidney disease (13 women, 15 men) and 28 with chronic kidney disease (13 women, 15 men) from the Department of Nephrology, Transplantology and Internal Diseases at the Pomeranian Medical University in Szczecin. Inclusion criteria were age ≥18 years and signed informed consent. For patient groups: Confirmed diagnosis of chronic kidney disease, based on KDIGO guidelines [41]. For control groups: No kidney, cardiovascular, or systemic inflammatory disease history.

### Material

Blood samples were drawn in the morning, after a fast, from the antecubital vein into two tubes: one with a clot activator to obtain serum, and the other with K3EDTA anticoagulant to obtain plasma. Both tubes were left at room temperature for 30 min and then centrifuged (10 min at room temperature, 1000 × *g*). Serum and plasma were then collected into separate tubes and frozen until use.

### Assays

Renalase levels were measured using three ELISA kits from different manufacturers: Cloud-Clone (Cloud-Clone Corp., Houston, TX, USA), BTLAB (Shanghai Korain Biotech Co., Ltd., Shanghai, China), and EIAab Life Sciences (EIAab Science Inc., Wuhan, China). The choice of assays was additionally informed by their relevance in the evolving renalase literature. Early studies following the initial description of renalase relied predominantly on selected commercially available ELISA platforms, whereas subsequent research introduced alternative manufacturers and assay designs. By including assays representing both earlier and more recent approaches, we aimed to capture methodological variability that may have contributed to discrepancies across studies.

The Cloud-Clone ELISA kit (SEC845Hu) is a sandwich enzyme immunoassay with a detection range of 3.12–200 ng/mL and LOD (limit of detection) of 12.5 ng/mL. According to the manufacturer’s recommendation, serum and plasma samples analysed with this assay were diluted 500-fold before measurement. The EIAab renalase ELISA (E1103h) kit is also a sandwich-type ELISA used to measure human Renalase (RNLS) in biological samples like serum, plasma, erythrocyte lysates, and urine, widely used in clinical research settings, especially in studying chronic kidney disease and haemodialysis patients, with a detection range of 0.78–50 ng/mL and LOD of 0.32 ng/mL. The BTLAB human renalase ELISA kit (E3109Hu) is designed to measure renalase concentration in serum, plasma, tissue/cell lysates, urine, and other biological fluids, with a detection range of 1–400 ng/mL and a LOD of 0.52 ng/mL. Each plasma and serum sample from the same tube was analysed in 3 technical replicates within the same assay. Measurements were performed in four analytical sessions conducted at weekly intervals. During each session, serum and plasma samples obtained from the same individuals were thawed once and analysed in parallel using all three ELISA assays by the same investigator. Each sample was measured in technical triplicate within a single 96-well plate for a given assay. To minimise potential batch effects, each analytical plate included samples from both the CKD and control groups. Due to the ongoing recruitment process, measurements were performed sequentially as participants were enrolled, resulting in a mixed distribution of study groups across analytical sessions. All kits were obtained from the same procurement batch, ensuring identical lot numbers across platforms. Each assay was calibrated using the manufacturer-provided standards according to the respective protocols. No common external reference standard was applied across platforms, as the study was designed to evaluate assay performance under routine laboratory conditions.

### EthicalQQQ approval

This study was conducted in accordance with the Declaration of Helsinki and was approved by the Local Bioethics Committee of Pomeranian Medical University (approval number: KB-006/13/23). Written informed consent was obtained from all participants before their inclusion in the study.

### StatisticalQQQ analysis

All statistical analyses were performed using Python (v3.11) and R (v4.2). Descriptive statistics were calculated for all continuous variables and are presented as mean ± standard deviation (SD) or median and interquartile range (IQR), depending on the distribution’s normality, assessed by the Shapiro-Wilk test.

Due to pronounced right skewness and heteroscedasticity in renalase concentrations across all ELISA platforms, log10 transformation was applied before agreement analyses and regression modelling. Bland–Altman analyses were conducted on log10-transformed values to assess proportional bias and the limits of agreement.

Group comparisons between CKD and control participants, as well as sex-based comparisons, were performed using two-sided independent *t*-tests or Mann–Whitney *U* tests, as appropriate. Paired serum–plasma comparisons were conducted using the Wilcoxon signed-rank test.

Spearman’s rank correlation coefficients were calculated to evaluate associations between renalase concentrations and clinical parameters (e.g. eGFR, creatinine, age, glucose, lipids, serum proteins). Correlation matrices were visualised as annotated heatmaps.

Binary logistic regression models were constructed to assess the predictive value of renalase concentrations for CKD status. For regression and Receiver operating characteristic (ROC) analyses, log10-transformed renalase values were used. ROC curves were generated, and area under the curve (AUC) values were calculated. Optimal thresholds were determined using the Youden index on the log10-transformed scale and subsequently back-transformed to the original concentration scale for reporting. These values represent model-derived statistical decision boundaries rather than directly measured analytical cut-offs.

All tests were two-tailed, and *p* < 0.05 was considered statistically significant.

## Results

Descriptive statistics of renalase concentrations measured using each assay and biological matrix are presented in Table 2. Substantial variability in renalase levels was observed across assays and study groups, with wide dispersion of values and differences in distribution characteristics depending on both the analytical platform and sample type. Given this variability, further analyses were performed to evaluate the agreement between renalase measurements obtained from serum and plasma.

**Table 2.** Descriptive statistics for renalase concentrations measured by three ELISA assays.

Assay	Matrix	Group	*n*	Mean	Median	Q1–Q3
BTLAB	Serum	Control	28	114.1	72.9	47.5–157.6
		CKD	28	56.0	47.9	36.6–54.1
	Plasma	Control	28	114.3	77.3	41.7–152.7
		CKD	28	58.8	44.2	39.1–58.4
Cloud-Clone*	Serum	Control	26	7422.5	4466.4	2939.3–8630.9
		CKD	25	12961.6	8646.3	3451.3–22384.2
	Plasma	Control	27	6925.3	3499.0	3006.2–8584.8
		CKD	23	13548.0	8613.9	4412.0–23434.2
EIAab	Serum	Control	19	16.6	0.9	0.4–17.3
		CKD	20	4.5	1.3	0.8–5.3
	Plasma	Control	28	20.6	4.1	1.4–19.2
		CKD	28	12.0	8.0	5.2–13.9

Values are expressed in ng/mL. *Cloud-Clone concentrations were recalculated to the original sample concentration using the manufacturer-recommended 500-fold dilution.

**Figure 1.**
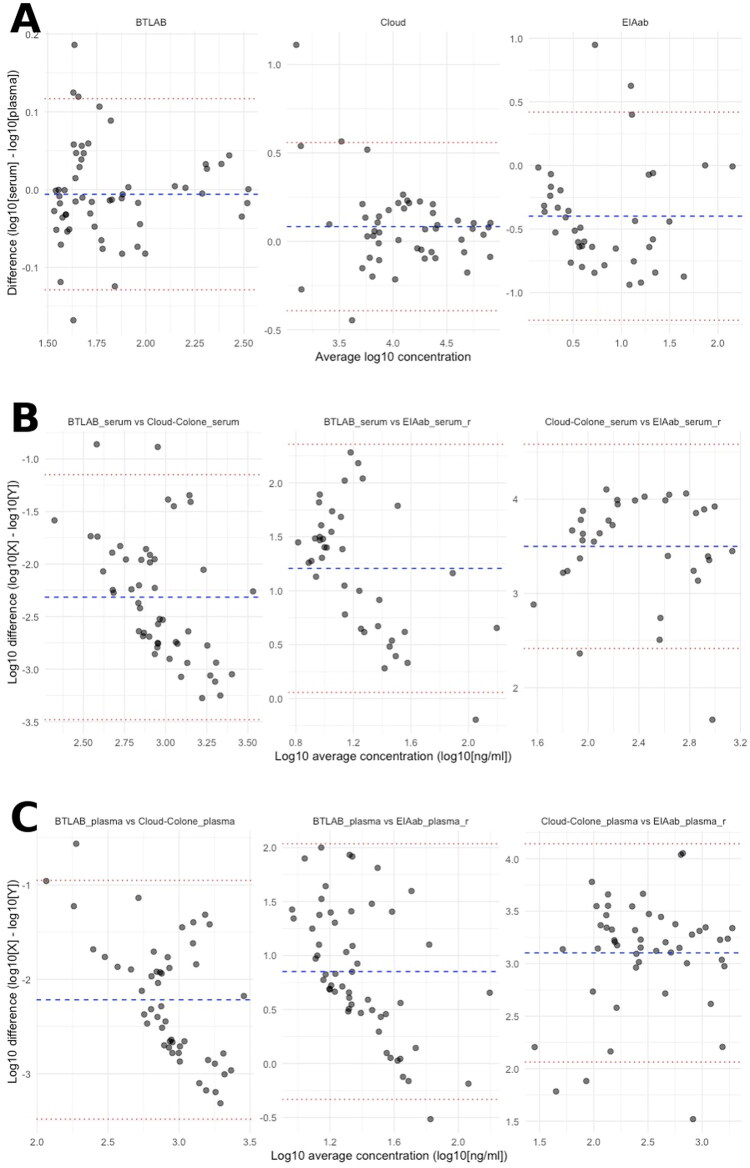
(A) Bland–Altman plots comparing serum and plasma renalase concentrations (log10 scale) across ELISA kits. (B) Bland–Altman plots comparing serum concentrations measured by different ELISA kits. (C) Bland–Altman plots for plasma samples.

Agreement between serum and plasma renalase concentrations varied by assay, as illustrated by Bland–Altman analysis (Figure 1) and summarised in Table 3. The BTLAB assay demonstrated the closest agreement between matrices, with a mean log10 bias of −0.006 and relatively narrow limits of agreement, indicating only minor differences between serum and plasma measurements. In contrast, the Cloud-Clone assay showed moderately higher renalase concentrations in serum than in plasma (mean log10 bias = +0.083) and wider limits of agreement. The EIAab assay exhibited the most pronounced matrix-related discrepancy, with substantially lower renalase concentrations measured in serum compared with plasma (mean log10 bias = −0.398) and broad limits of agreement, suggesting limited interchangeability of results obtained from the two matrices.

**Table 3.** Comparison of renalase concentrations between serum and plasma across different ELISA kits.

Method (manufacturer)	Mean difference (log10)	Percent difference (serum vs plasma)	SD of differences	LoA lower	LoA upper	Sample size (*n*)	Outliers (out of LoA)
BTLAB	–0.006	–1.4	0.063	–0.129	+0.117	56	4
Cloud-Clone	+0.083	+21.1	0.243	–0.393	+0.559	49	3
EIAab	–0.398	–60.0	0.418	–1.220	+0.420	39	2

A positive mean log-difference indicates higher renalase concentrations in serum than in plasma, whereas a negative value indicates higher concentrations in plasma.

Direct comparison of renalase concentrations obtained using different ELISA platforms revealed substantial systematic differences between assays (Tables 4 and 5). For serum samples, renalase values measured with the BTLAB assay were markedly lower than those obtained with the Cloud-Clone assay (mean log10 difference = −2.32). In contrast, BTLAB measurements were substantially higher than those obtained with the EIAab assay (mean log10 difference = +1.21). The largest discrepancy was observed between the Cloud-Clone and EIAab assays, with Cloud-Clone yielding renalase concentrations several orders of magnitude higher on average (mean log10 difference = +3.50). A similar pattern of inter-assay variability was observed for plasma samples (Table 4), confirming that the magnitude of analytical differences between platforms was not restricted to a single biological matrix.

**Table 4.** Bland–Altman comparison of the analysed ELISA tests.

Comparison pair	Mean difference (log10)	Percent difference (method X vs Y)	SD (log10)	LoA Lower	LoA Upper	*n*	Outliers
BTLAB vs Cloud-Clone	–2.32	–99.95	0.595	–3.48	–1.15	51	2
BTLAB vs EIAab	+1.21	+1,521	0.587	+0.06	+2.36	39	1
Cloud-Clone vs EIAab	+3.50	+31,622	0.552	+2.42	+4.58	34	2

Positive values indicate higher renalase concentrations in the first-listed assay, whereas negative values indicate higher concentrations in the second-listed assay.

**Table 5.** Bland–Altman for plasma samples.

Comparison pair	Mean difference (log10)	Percent difference (method X vs Y)	SD (log10)	LoA lower	LoA upper	*n*	Outliers
BTLAB vs Cloud-Clone	–2.22	–99.40	0.645	–3.48	–0.95	50	1
BTLAB vs EIAab	+0.851	+610.0	0.604	–0.33	+2.04	55	1
Cloud-Clone vs EIAab	+3.10	+11,580	0.530	+2.06	+4.14	49	3

Positive values indicate higher renalase concentrations in the first-listed assay, whereas negative values indicate higher concentrations in the second-listed assay.

### Comparison of renalase concentrations between control and CKD groups

**Figure 2.**
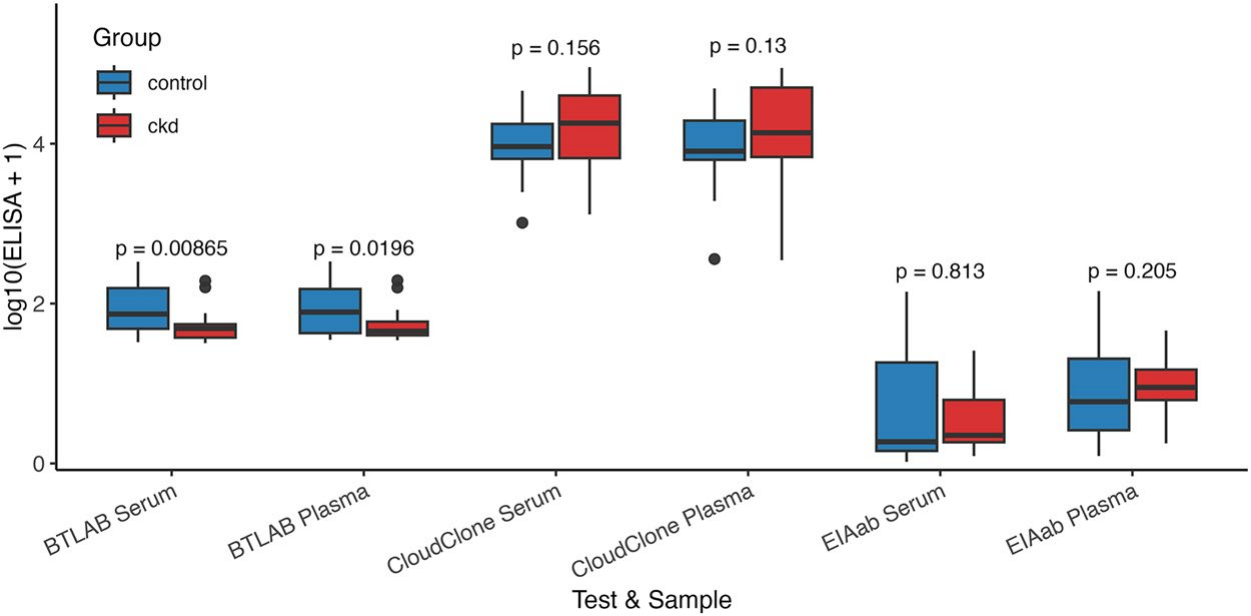
Boxplots of log-transformed renalase levels across test platforms and sample types. Significant group differences (CKD vs. control) were observed for BTLAB serum (*p* = 0.0094) and plasma (*p* = 0.0204).

Renalase concentrations in serum and plasma samples were compared between individuals with chronic kidney disease (CKD) and healthy controls using three different ELISA kits (BTLAB, Cloud-Clone, and EIAab). The distribution of log-transformed renalase concentrations across study groups and assay platforms is shown in Figure 2. Due to the non-normal distribution of renalase levels, the non-parametric Mann–Whitney *U* test was used for all comparisons.

Using the BTLAB ELISA, significant differences in renalase concentrations were observed between the CKD and control groups in both serum (*U* = 551.0, *p* = 0.0094) and plasma (*U* = 534.0, *p* = 0.0204). In both cases, renalase concentrations were significantly lower in the CKD group compared with controls, suggesting a potential association between reduced BTLAB-measured renalase levels and impaired kidney function.

In contrast, the Cloud-Clone ELISA did not reveal statistically significant differences between groups. The comparisons in serum (*U* = 249.0, *p* = 0.1549) and plasma (*U* = 232.0, *p* = 0.1289) indicated trends towards increased renalase in CKD, but these did not reach significance.

Similarly, the EIAab ELISA showed no significant difference in serum renalase levels between CKD and control subjects (*U* = 181.0, *p* = 0.8112) and in plasma (*U* = 302, *p* = 0.2037), indicating considerable variability in detection performance among the tested ELISA methods.

### Sex-based differences in renalase concentrations

To investigate potential sex-related differences in circulating renalase concentrations, comparisons between females and males were performed across the three ELISA platforms, both biological matrices, and study groups using the non-parametric Mann–Whitney U test. In the control group, no statistically significant sex-related differences were observed for any assay or sample type. For example, in the BTLAB assay, serum renalase concentrations were 88.55 [45.58–208.92] ng/mL in females and 63.41 [48.20–85.05] ng/mL in males (*U* = 112.0, *p* = 0.519), while plasma concentrations were 92.13 [42.03–193.68] ng/mL and 65.50 [41.40–97.51] ng/mL, respectively (*U* = 113.0, *p* = 0.490). Similar non-significant findings were observed for the Cloud-Clone and EIAab assays (all *p* > 0.17).

In contrast, sex-related differences were observed in the CKD group in an assay-dependent manner. Using the Cloud-Clone ELISA, renalase concentrations were significantly higher in males than in females in both serum (40.01 [15.66–72.38] vs 10.01 [4.43–21.66] ng/mL; *U* = 36.0, *p* = 0.024) and plasma (50.33 [10.79–67.52] vs 11.15 [2.60–18.40] ng/mL; *U* = 30.0, *p* = 0.029). Likewise, plasma renalase concentrations measured with the EIAab assay were higher in males than in females (10.83 [6.42–32.81] vs 6.10 [3.22–10.50] ng/mL; *U* = 51.0, *p* = 0.034). No statistically significant sex-related differences were detected using the BTLAB assay in either serum (49.92 [36.13–66.46] vs 45.97 [36.66–51.00] ng/mL; *U* = 78.0, *p* = 0.381) or plasma (52.95 [40.13–71.43] vs 43.40 [38.50–47.10] ng/mL; *U* = 64.0, *p* = 0.128), nor in serum measured with the EIAab assay (3.82 [0.96–7.60] vs 0.96 [0.74–1.28] ng/mL; *U* = 30.0, *p* = 0.181). Overall, the direction and magnitude of sex-related differences varied by assay.

### Correlation between renalase levels and biochemical parameters

**Figure 3.**
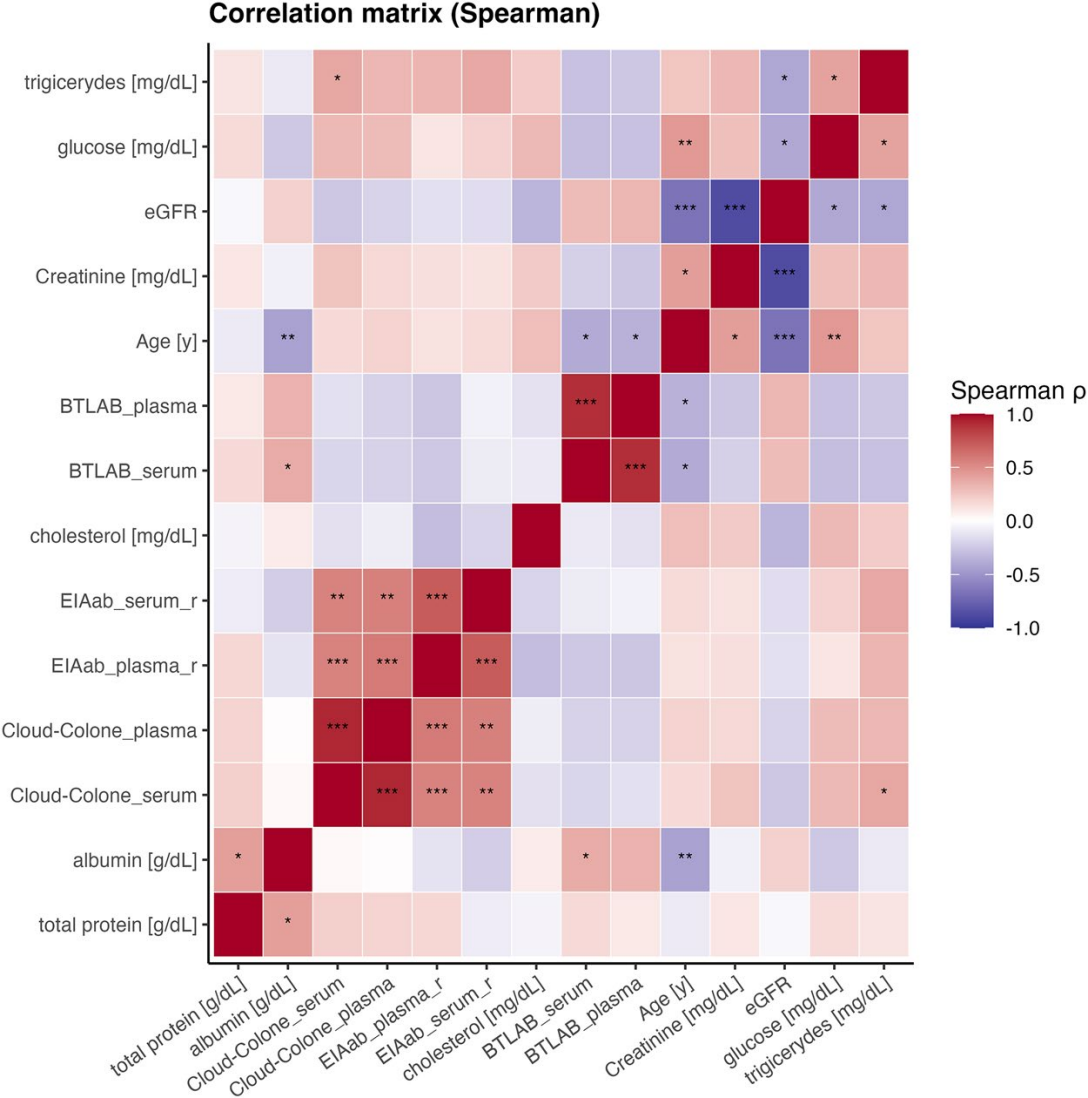
Correlation heatmap between clinical variables (age, eGFR, creatinine, glucose, cholesterol) and biomarker levels from different assays (ElAab, BTLAB, Cloud-Clone).

Spearman correlation analysis was conducted to assess associations between renalase concentrations—measured in serum and plasma using three ELISA kits (EIAab, BTLAB, and Cloud-Clone)—and selected clinical and biochemical parameters, including age, estimated glomerular filtration rate (eGFR), creatinine, total protein, albumin, glucose, cholesterol, and triglycerides (Figure 3).

In the EIAab ELISA, serum renalase levels showed no statistically significant correlations with age, eGFR, or albumin. However, a significant positive correlation was observed with triglyceride levels (ρ = 0.39, *p* = 0.0202). In plasma, renalase levels were significantly negatively correlated with cholesterol (ρ = −0.30, *p* = 0.0281) and positively correlated with triglycerides (ρ = 0.33, *p* = 0.0173).

In the BTLAB ELISA, serum renalase levels showed significant negative correlations with age (ρ = −0.39, *p* = 0.0031) and glucose (ρ = −0.30, *p* = 0.0289), and a positive correlation with albumin (ρ = 0.37, *p* = 0.0064) and eGFR (ρ = 0.30, *p* = 0.0442). Plasma renalase concentrations showed a similar pattern, with significant negative correlations with age (ρ = −0.36, *p* = 0.0062) and glucose (ρ = −0.28, *p* = 0.0396), and positive correlations with albumin (ρ = 0.34, *p* = 0.0124) and eGFR (ρ = 0.32, *p* = 0.0286).

Renalase levels measured by the Cloud-Clone ELISA were significantly positively correlated with glucose (serum: ρ = 0.31, *p* = 0.0273; plasma: ρ = 0.29, *p* = 0.0381) and triglycerides (serum: ρ = 0.39, *p* = 0.0047; plasma: ρ = 0.32, *p* = 0.0243), but not with markers of kidney function or protein metabolism. An additional analysis confirmed the expected age-related decline in renal function. In the control group, a statistically significant negative correlation was observed between age and eGFR (ρ = −0.59, *p* = 0.0096). In the CKD group, a similar trend was observed (ρ = −0.34), although it did not reach statistical significance (*p* = 0.0777), potentially due to reduced variability within the diseased cohort.

### Association of renalase levels with age and renal function

Among the three ELISA platforms used in this study, the BTLAB assay showed the most consistent and statistically significant associations of renalase levels with patient age and renal function as measured by eGFR (Figure 3). Specifically, renalase concentrations measured with the BTLAB kit were negatively correlated with age in both serum (ρ = −0.39, *p* = 0.0031) and plasma (ρ = −0.36, *p* = 0.0062), and positively correlated with eGFR in both sample types (serum: ρ = 0.30, *p* = 0.0442; plasma: ρ = 0.32, *p* = 0.0286). In contrast, neither the EIAab nor the Cloud-Clone assays showed statistically significant correlations between renalase and eGFR, and their associations with age were weak and non-significant.

### Diagnostic performance of renalase ELISAs in predicting CKD

**Figure 4.**
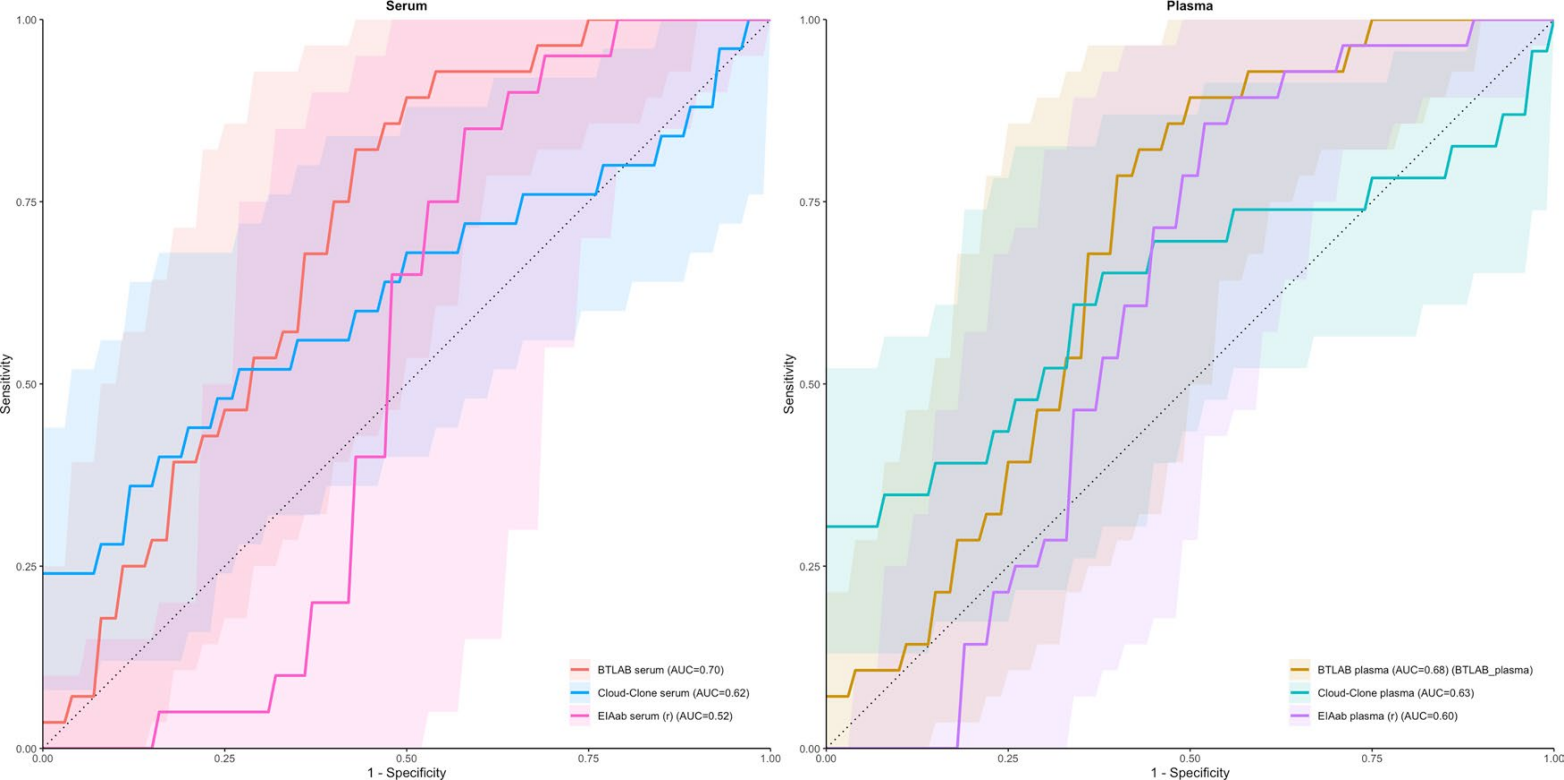
ROC curves for the diagnostic performance of three ELISA platforms.

To assess whether circulating renalase concentrations can differentiate patients with chronic kidney disease (CKD) from healthy controls, logistic regression models were constructed using log10-transformed values from three commercial ELISA platforms (BTLAB, Cloud-Clone, and EIAab). ROC analysis with Youden index optimisation was then used to compare discriminatory performance across assays. As illustrated in Figure 4, renalase concentrations measured with the BTLAB assay showed the highest discriminatory ability in both serum (AUC = 0.70) and plasma (AUC = 0.68). The Cloud-Clone assay performed less well (serum AUC = 0.62; plasma AUC = 0.63), and the EIAab assay performed even worse (serum AUC = 0.52; plasma AUC = 0.60), indicating notable assay-dependent differences in renalase-based group discrimination. Threshold values derived from ROC analysis were exploratory and based on log10-transformed data; therefore, they should not be interpreted as directly measurable clinical cut-offs or as validated clinical decision limits.

### Comparison of renalase concentrations between serum and plasma

To assess whether the sample matrix influences renalase quantification, paired measurements of renalase concentrations in serum and plasma were compared for each ELISA assay using the Wilcoxon signed-rank test. Bland–Altman analysis assessed agreement and interchangeability between serum and plasma measurements, and the following comparisons evaluated whether systematic differences between matrices reached statistical significance at the group level. For the BTLAB ELISA, no significant difference was observed between serum and plasma concentrations (*W* = 672.0, *n* = 56, *p* = 0.304), indicating consistent performance across sample types. In contrast, significant differences were identified for both the Cloud-Clone and EIAab assays. The Cloud-Clone ELISA showed a modest but statistically significant discrepancy between serum and plasma (*W* = 362.0, *n* = 49, *p* = 0.012), whereas the EIAab ELISA exhibited a pronounced matrix effect (*W* = 87.0, *n* = 39, *p* < 0.001), with systematically divergent values.

## Discussion

In this study, we systematically evaluated the performance of three commercially available ELISA kits—BTLAB, Cloud-Clone, and EIAab—for quantifying circulating renalase (RNLS) in serum and plasma from individuals with and without chronic kidney disease (CKD). Our findings reveal substantial inter-assay variability, matrix-dependent discrepancies, and divergent diagnostic performance, underscoring the methodological challenges inherent in using ELISA-based quantification of renalase as a clinical or research tool.

Marked inconsistencies were observed across ELISA kits. Bland–Altman analyses of log-transformed data revealed substantial differences in renalase concentrations across assays. For instance, BTLAB versus Cloud-Clone showed a mean log-difference of −2.32, corresponding to a 99.95% lower concentration reported by BTLAB. Similarly, Cloud-Clone values were, on average, >30,000% higher than EIAab, with wide limits of agreement and frequent outliers. These discrepancies suggest that the three ELISAs are not interchangeable, likely due to differences in antibody specificity, epitope recognition, and calibration standards. Such assay-dependent variability is well documented in the immunoassay literature. Studies have shown that even assays targeting the same analyte can yield divergent results due to heterogeneity in reagent design and the lack of standardised calibrators [16]. The absence of a certified reference standard for renalase further compounds this issue. In addition to methodological factors, the biological heterogeneity of circulating renalase may also contribute to assay-dependent variability. Chang et al. [11] reported distinct measurable forms of renalase in human plasma, suggesting that differential epitope exposure or protein processing may influence antibody-based detection. While that study focused primarily on the biochemical characterisation of renalase fractions, our findings provide additional clarification by demonstrating that, under routine analytical conditions, the selection of commercially available ELISA platforms alone may substantially affect measured concentrations, matrix agreement, and apparent diagnostic performance.

Despite its widespread use, ELISA is known for several methodological limitations, including susceptibility to matrix effects, limited dynamic range, cross-reactivity, and batch-to-batch variability [18]. In our study, serum-plasma comparability further highlighted assay fragility: while BTLAB showed consistent measurements across matrices (*p* = 0.304), both Cloud-Clone and EIAab exhibited statistically significant matrix effects (*p* < 0.05) with pronounced directional biases. Without transparent performance metrics or inter-laboratory standardisation, confidence in the clinical applicability of these assays remains limited.

Only the BTLAB assay revealed significant differences in renalase concentrations between CKD patients and controls (*p* < 0.05) in both serum and plasma, suggesting that this platform is more sensitive to CKD-related alterations in circulating renalase. Moreover, BTLAB-derived renalase levels showed biologically plausible associations with renal and metabolic parameters, including positive correlations with estimated glomerular filtration rate (eGFR) and albumin, and negative correlations with age and glucose, consistent with previous reports [21,42–44]. In contrast, renalase concentrations measured using the EIAab and Cloud-Clone assays were not significantly associated with renal function in the present cohort. Earlier studies employing the EIAab platform have reported higher renalase levels in CKD populations; however, in our dataset, the magnitude and direction of group differences were less consistent. Such discrepancies may reflect differences in study populations, biological matrices analysed, and analytical workflows rather than inherent limitations of the assay itself. Notably, renalase levels measured with these platforms were mainly associated with metabolic markers such as triglycerides and glucose, which may suggest greater susceptibility to matrix effects or to the analytical context.

Importantly, no consistent sex-related differences were observed among healthy controls, although some sex-specific patterns were noted in CKD patients, but only with selected ELISA platforms. This finding should be interpreted with caution. Chronic kidney disease is associated with persistent systemic inflammation, oxidative stress, immune activation, and marked alterations in plasma protein composition, all of which may influence the analytical performance of antibody-based assays. Endogenous immunoglobulins, including heterophilic antibodies or rheumatoid factor-like molecules, have been shown to interfere with sandwich immunoassays by forming non-specific bridges between capture and detection antibodies, potentially leading to falsely elevated or suppressed analyte concentrations [15,16,18,45]. Importantly, the prevalence and clinical relevance of immunoassay interference may be increased in chronic inflammatory conditions and in populations with altered immune status, including patients with advanced kidney disease [18]. Moreover, sex-related biological differences in CKD have been widely documented. These include differences in immune response, hormonal regulation, body composition, and patterns of renal disease progression, which may contribute to variability in circulating protein profiles [46,47]. These pathophysiological mechanisms may interact with assay-specific characteristics such as antibody epitope recognition, avidity, or susceptibility to matrix effects. The absence of consistent sex-related differences when renalase was measured using the analytically most stable ELISA platform in the present study suggests that at least part of the observed variability may reflect assay-dependent analytical sensitivity rather than true biological dimorphism. Similar assay-dependent discrepancies have been reported for other circulating biomarkers measured using immunoassays, particularly in heterogeneous clinical populations characterised by systemic inflammation or altered plasma protein composition [16,45]. Therefore, sex-specific differences in renalase concentrations detected only with selected assay platforms should be interpreted within the broader framework of immunoassay variability and CKD-related pathophysiological complexity.

Using ROC analysis to assess diagnostic performance, the BTLAB assay showed the highest discriminatory ability among the evaluated platforms, with moderate performance in both serum and plasma. In contrast, the Cloud-Clone and EIAab assays demonstrated weaker discrimination, further emphasising the assay-dependent nature of renalase-based group separation. Importantly, the less favourable balance between sensitivity and specificity observed for the other platforms suggests that different assays may introduce distinct patterns of misclassification, further limiting their clinical applicability. Overall, these findings indicate that the apparent diagnostic value of circulating renalase is highly dependent on the assay used and should be interpreted with caution until methodological standardisation is achieved.

Collectively, our results underscore the need for rigorous assay validation before adopting renalase as a clinical biomarker. Among the evaluated assays, BTLAB showed the most consistent analytical performance in this cohort, though further external validation is required. The substantial discrepancies observed across ELISA platforms reflect broader challenges in translational nephrology, where the lack of assay standardisation continues to hinder biomarker development. This issue is compounded by the fact that the terms *serum* and *plasma* are sometimes used interchangeably in the renalase literature, potentially contributing to inconsistencies in interpretation and cross-study comparisons [48]. Additionally, the lack of internationally accepted reference material for renalase is an important limitation for both the present study and the broader biomarker field, as it currently prevents meaningful calibration harmonisation across analytical platforms and complicates the interpretation and comparability of published findings.

Despite these limitations, our findings underscore renalase’s translational relevance. Once standardised and validated, renalase could be incorporated into CKD risk stratification models, assist clinicians in monitoring disease progression, and potentially guide treatment decisions, especially when used alongside established biomarkers such as NGAL, cystatin C, or KIM-1. As with these biomarkers, moving renalase from bench to bedside will require harmonised protocols, reference standards, and multicentre validation studies. Such efforts could unlock renalase’s full potential as a clinically useful biomarker in nephrology.

## Data Availability

The data that support the findings of this study are available from the corresponding author, NS, upon reasonable request.
